# Can Score Databanks Help Teaching?

**DOI:** 10.1371/journal.pone.0015695

**Published:** 2011-01-05

**Authors:** Vitor Rosa Ramos de Mendonça, Bruno Bezerril Andrade, Alessandro Almeida, Manoel Barral-Netto

**Affiliations:** 1 Faculdade de Medicina, Universidade Federal da Bahia, Salvador, Bahia, Brazil; 2 Centro de Pesquisas Gonçalo Moniz, Fundação Oswaldo Cruz, Salvador, Bahia, Brazil; 3 Instituto de Investigação em Imunologia, Instituto Nacional de Ciência e Tecnologia, São Paulo, Brazil; Aga Khan University, Pakistan

## Abstract

**Background:**

Basic courses in most medical schools assess students' performance by conferring scores. The objective of this work is to use a large score databank for the early identification of students with low performance and to identify course trends based on the mean of students' grades.

**Methodology/Principal Findings:**

We studied scores from 2,398 medical students registered in courses over a period of 10 years. Students in the first semester were grouped into those whose ratings remained in the lower quartile in two or more courses (low-performance) and students who had up to one course in the lower quartile (high-performance). ROC curves were built, aimed at the identification of a cut-off average score in the first semesters that would be able to predict low performances in future semesters. Moreover, to follow the long-term pattern of each course, the mean of all scores conferred in a semester was compared to the overall course mean obtained by averaging 10 years of data. Individuals in the low-performance group had a higher risk of being in the lower quartile of at least one course in the second semester (relative risk 3.907; 95% CI: 3.378–4.519) and in the eighth semester (relative risk 2.873; 95% CI: 2.495–3.308). The prediction analysis revealed that an average score of 7.188 in the first semester could identify students that presented scores below the lower quartiles in both the second and eighth semesters (p<0.0001 for both AUC). When scores conferred by single courses were compared over time, three time-trend patterns emerged: low variation, upward trend and erratic pattern.

**Conclusion/Significance:**

An early identification of students with low performance may be useful in promoting pedagogical strategies for these individuals. Evaluation of the time trend of scores conferred by courses may help departments monitoring changes in personnel and methodology that may affect a student's performance.

## Introduction

Medical schools are responsible for preparing undergraduate students to become professionals. In this context, a powerful connection between teaching and learning is essential for an adequate education. Most medical schools assess students' performance by conferring scores. Recent studies have reported the use of large score databanks to infer future professional performance [1]. Although the association between performance in medical courses at school and professional performance is controversial [2], a relationship has been found between the scores of medical students in basic medical science courses taken in the first year of medical school and the subsequent performance of these students during and beyond medical school [3]. Thus, student performance in early stages of medical education could be a good predictor of their overall success in other courses. Furthermore, many medical students commonly present learning difficulties and need some pedagogical intervention to improve their performance. Identifying these students at early stages of their medical studies may invite extra attention or necessary efforts to improve their performance. Students' scores must also discriminate between students who need supplemental effort for their education and those who do not in order to achieve their maximum analytical potential [3].

Each medical course develops its own evaluation system for students. Different assessment patterns could impact the need for changes in teaching methods over time. Even if not intended, evaluation performance may vary according to substitution of teachers throughout the years and also the role of each teacher in a given course. The scores conferred in the same course over time may help to discriminate intended from unintended changes in teaching methodology and/or evaluation systems. It may also help to identify external factors that may influence student performance. Properly conducted overall student assessment is a great tool to highlight student skills. An ideal evaluation system should be as precise as possible to measure differences among students at different levels of achievement [4].

The aim of this study is to use a large score databank to identify students with low performance in the early semesters of medical education. These students may benefit from special attention to their needs to improve learning and yield better results on their scores. Additionally, this study performs an analysis of the pattern of mean scores of each course over a period of 10 years, aimed at identifying the trend for each course. This approach may help the department involved to evaluate the changes introduced during the semester. These findings may be important for guiding individualized assistance for most students who need help. They may also suggest innovative educational polices to improve overall performance in the medical course.

## Methods

In this report we present an observational, retrospective study from 1994 to 2003 of all students regularly registered in the mandatory courses of the curriculum of the School of Medicine, Federal University of Bahia (UFBA), which is a very traditional Brazilian medical school that registers 80 new medical students per semester. Within this medical school, the assessment of student performance is registered using scores from zero to ten, which are stored in the collegiate databank. The data collected consists of the registration numbers and scores in courses taken between the first semester and eighth semester, which is the final period before the four semesters of medical internship. The study identification number allowed for the identification of all the scores obtained from a single individual in each course without revealing the name of the student. Since an individual informed consent was not obtainable, university authorities' permission was sought and granted after an ethics analysis.

Medical courses in Brazil last for an average of twelve semesters. The first eight are intended for theory-practice learning. The basic science subjects are concentrated in the first period. The last four semesters are performed as supervised practice, known as surgical and clinical rotations, in different medical specialties. During the first, fifth, sixth and seventh semesters students are enrolled in four courses per semester; in the second, third and eighth semesters there are five courses in each one; three courses comprise the fourth semester. Every course lasts for one single semester. Within the medical school in which the present study was performed, the same method of assessment is used to evaluate all the eight semesters in the medical school. Such method is based on theoretical open written questions or objective multiple-choice questions. In those courses in which some degree of practical activity is a matter of assessment, such as anatomy or histology, standardized practical evaluations are also performed, however with lower impact in the final score. During the period of this study, the same style of assessment was maintained, as well as there were no changes in the medical curriculum.

During the study period, a total of 2,398 medical undergraduate students were registered in mandatory courses from the initial eighth semesters. The first objective of the present study was to test the prediction of the students' academic performance in the second and eighth semesters using scores obtained in the first semester. Thus, all the students who had scores from the first, second and eighth semesters registered in the score databank within the years of the study were included. This sub-sample totalized scores from 1,071 students (44.66% of all the medical students registered). The cohorts of students followed up have been selected by the same style of admission tests to start graduation, so changes in the student body were negligible. In addition, the mean summated scores of students in each academic year for a summation of all courses/semesters were relatively stable, ranging from 6.5 to 7.0. We tested correlations between the mean scores of these students in the first semester and their scores in the second or eighth semester to demonstrate if the performance in the beginning was consistent with the performance in the remainder of the course. The correlations were calculated using the Pearson coefficient. The students were grouped according to their scores into two categories based on the possible need for individual assistance. Students whose ratings remained in the lower quartile in two or more courses were classified as the low-performance group and those who had no courses or only one course in the lower quartile were nominated for the high-performance group. Students in each of these groups had their performance evaluated in the second and eighth semesters to assess the correlation between student performances at the beginning and at the end of the theoretical part of medical studies. Therefore, linear regression was used for the estimation of the causal relation (relative risk) between the performances in the first and second or eighth semesters.

In an additional approach, we calculated the cut-off values for the scores obtained in the first semester that could predict a low performance in the second or eighth semesters using Receiver-Operator Characteristics (ROC) curves with C-statistics. In an attempt to build the ROC curves, we estimated the students' performances in each semester by averaging the scores from the courses of each period. The cut-off values represent the score mean in the first semester that presents the highest sensitivity, specificity and likelihood ratio for predicting a low performance in the second or eighth semesters (scores within the lower quartile).

**Figure 1:**
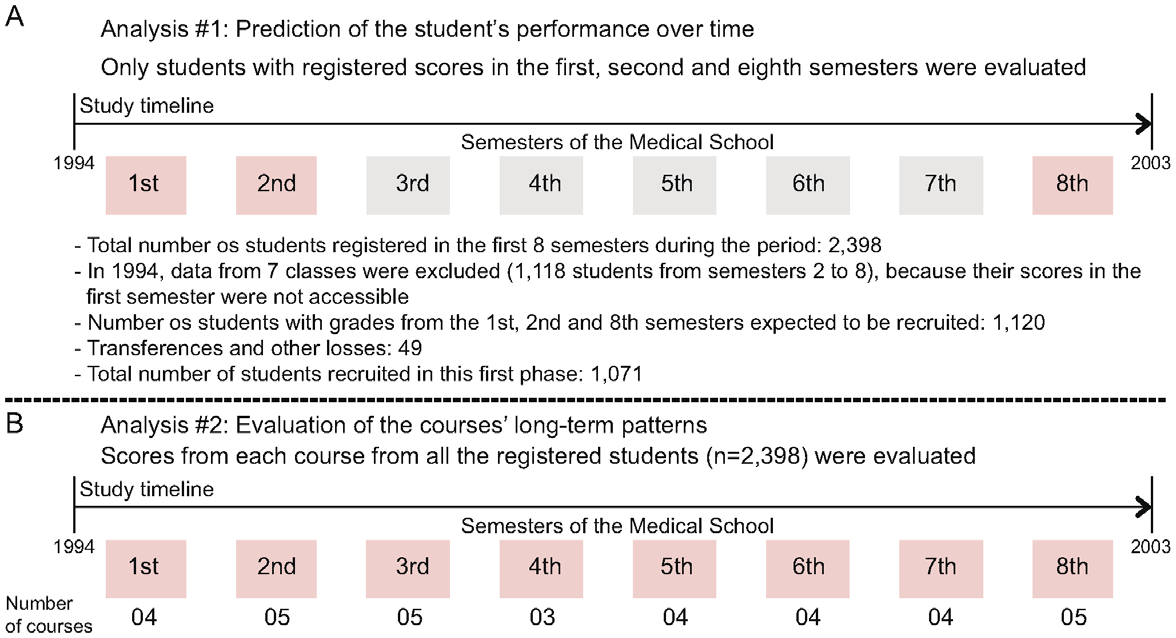
Summary of the sampling methods used in the study. A total of 2,398 students were registered in the medical school within the period of the study, from 1994 to 2003. Two different approaches were used to assess the two major objectives of the study. (A) In order to predict the academic performance over time, only students with registered scores in the first, second and eight semesters from 1994 until 2003 were included (n = 1,071). (B) With an attempt to evaluate the different courses' long-term patterns, mean scores from all the students in each course (n = 2,398) was calculated in each year of the study period.

The second objective of this study was to follow each course's long-term pattern, the mean of scores conferred in a semester was compared to the overall course mean (±1 standard deviation, SD) over 10 years. Hence, data from all the 20 registered classes were included (n = 2,398), as this additional approach does not require follow up of a specific student. A summary of the sampling approaches used for the different analyses are shown in the Figure 1. All data were analyzed using the GraphPad Prism 5.00 (GraphPad software, San Diego, CA). Differences were considered significant at p<0.05.

## Results

**Figure 2:**
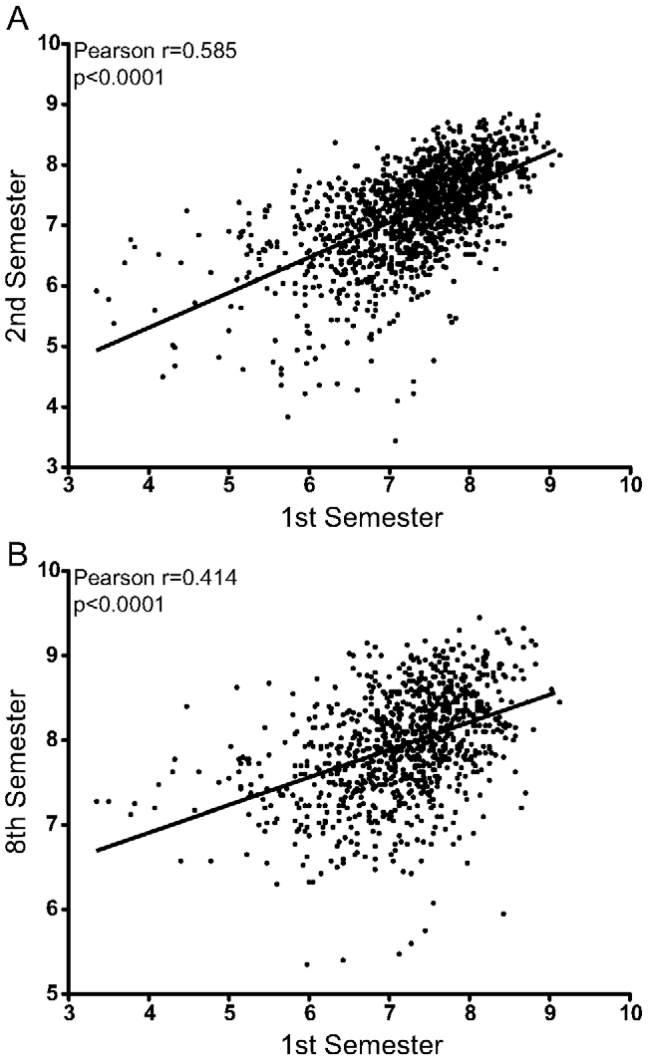
Correlation between scores of the students from the first and second or eighth semesters. The global score obtained from each student in the first semester of medical school was correlated with the scores obtained in the second (A) or eighth (B) semesters using the Pearson coefficient (n = 1,071). Linear regression was used to illustrate the general trend of the correlations. The p and r values are plotted in each graph.

Considering all the registered students, there was a strong and statistically significant correlation between the mean scores obtained by students in their first semester and those in the second semester (Figure 2A; r = 0.5851, 95% CI:0.5507–0.6175; p<0.0001). Interestingly, there was a positive correlation between the scores in the first semester and those in the eighth semester (Figure 2B; r = 0.4144, 95% CI:0.3616–0.4647; p<0,0001).

**Figure 3:**
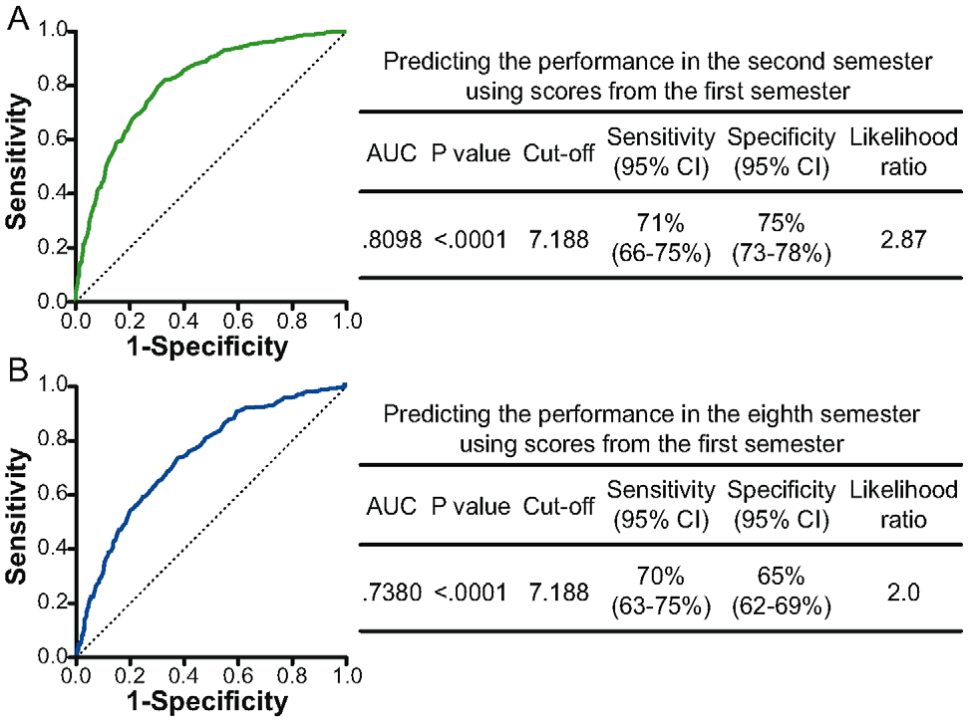
Establishing a cut-off grade in the first semester to predict students' performances in the second or eighth semesters. ROC curves were built to identify a cut-off grade in the first semester that can predict the students' performances in the second (A) or in the eighth (B) semesters. The cut-off value represents the average grade in the first semester that presented the higher sensitivity and specificity with a considerable likelihood ratio to predict that the further grades will be place in the lower quartile in the subsequent semesters. C-statistics are illustrated next to the curves and were used to verify the validation of the ROC curves and to establish the predictive power of the cut-off grade. AUC, area under the curve; CI, confidence interval.

We decided then to evaluate on an individual basis the relationship between the performance in the first semester to that in the second and the eighth semesters in terms of the relative performance of other students in the same period. For such an assessment we considered two groups of students: those who stayed in the three highest quartiles in all or all-but-one course, and those who remained in the lowest quartile in two or more courses. The performance of medical students in their first semester of the Medical School correlated well to their performance in the second or eighth semesters, as shown in Table 1. Individuals whose performance appeared in the lower quartile in at least two courses of the first semester had a high risk of being in the lower quartile of at least one course in the second semester, with a relative risk of 3.907 (95% CI: 3.378–4.519). Furthermore, students who were in the lower quartile in their first semester also had a higher risk of being in the lower quartile in the eighth semester with a relative risk of 2.873 (95% CI: 2.495–3.308). An additional analysis using ROC curves revealed that an average grade in the first semester of 7.188 can serve as a good cut-off value to predict a low performance in the second semester (Area Under Curve: 0.8098; sensitivity: 71%; specificity: 75%; likelihood ratio: 2.87; p<0.0001) and also in the eighth semester (Area Under Curve: 0.7380; sensitivity: 70%; specificity: 65%; likelihood ratio: 2.0; p<0.0001) (Figure 3A and 3B).

**Table 1:** Prediction of student grade performance in later semesters of medical study using scores from the first semester.

	2nd semester			8th semester		
1st semester	Upper quartilesn (%)	Lower quartile in >1 coursen (%)	Relative risk (95%CI)	Upper quartilesn (%)	Lower quartile in >1 coursen (%)	Relative risk (95%CI)
Lower quartile in 0-1 course	567(91.45)	157(34.81)		541(85.06)	183(42.07)	
			3.91*(3.38–4.52)			2.87*(2.49–3.31)
Lower quartile in >1 course	53(8.55)	294(65.19)		95(14.94)	252(57.93)	

Linear regression was performed to estimate the causal effect of the performance on the first semester on the second or eighth semesters. A total of 1,071 students registers were used. 95%CI: 95% confidence interval.

*p<0.05.

**Figure 4:**
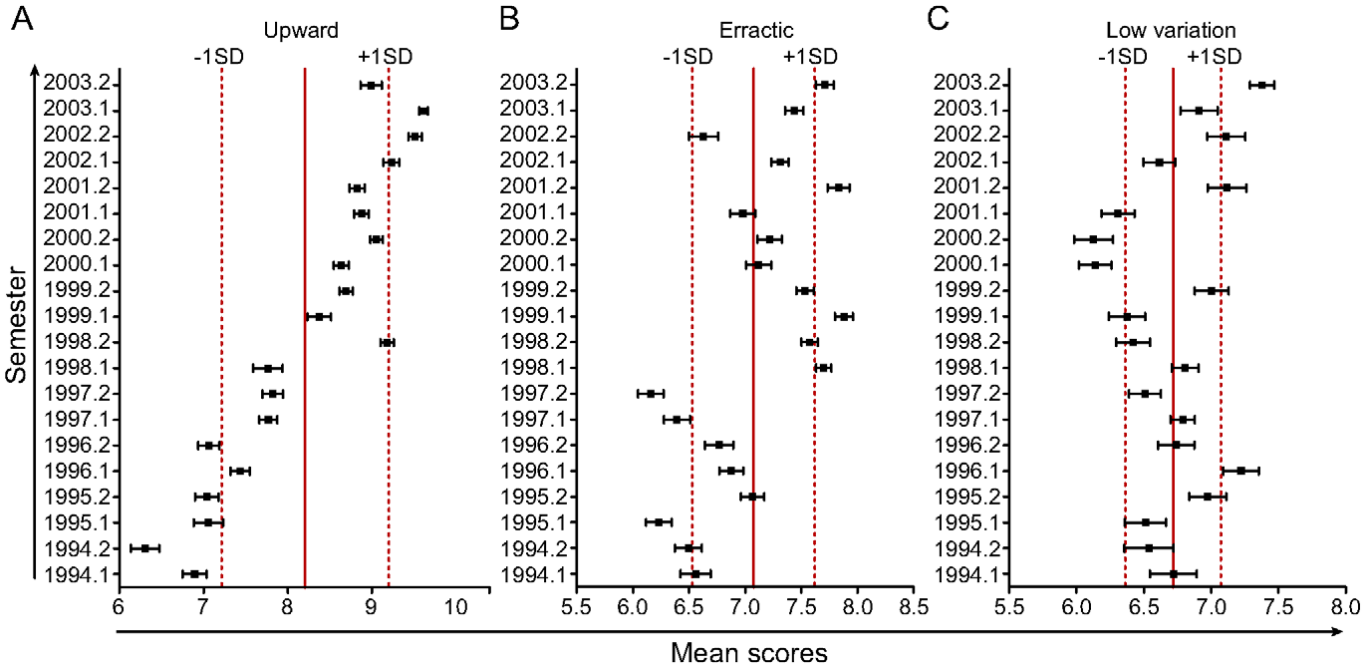
Scores distribution in different types of course assessments. A retrospective observation was performed from 1994 to 2003, evaluating grades from all students regularly registered in the mandatory courses from the curriculum of the School of Medicine, Federal University of Bahia (UFBA). Three major patterns of grade distribution were noted and representative courses were used to illustrate the differences. Thus, some courses presented growth assessment over time (upward trend; A) while others presented oscillating (erratic trend; B) or regular (low variation trend; C) assessments during the period of the study. Full vertical lines represent the global means for each course. Dashed lines represent ±1 standard deviation (SD). Dots and whiskers represent the mean grade and standard error of each semester.

When scores conferred by single courses were compared over a period of 10 years (most with a total of 13 groups of students), three time-trend patterns emerged. A group of courses had a consistent upward trend in mean scores conferred, as shown in Figure 4A. Moreover, in another group of courses, no clear pattern was noted (erratic trend; Figure 4B). It is noteworthy that no downward trend was observed. Yet in another group of courses, very low variations in terms of the mean of attributed scores were exhibited (Figure 4C).

Finally we assessed the courses in which students failed in the second and eighth semesters when they had failed in the first semester. The students identified as having a low performance in the first semester failed in different courses in the second and eighth semesters, with no clear pattern. These courses from the second and eighth semesters presented no direct relationships and the assessments were also not similar, with some courses presenting the low variation pattern while other presented erratic or upward trends. This observation suggests that the students with low performance do not have a specific deficiency with one aspect of a certain subject.

## Discussion

Upon entering medical school, students face a new academic environment that requires a new way of life, as well as time and dedication. However, many cannot adapt to this change and end up performing poorly. The present report shows that low performance in the first semester serves as an indicator of lower achievement in later semesters of the medical course. Therefore, identification of students with learning difficulties is essential to provide them with special pedagogical attention.

Frequency of attendance was related to academic performance in an introductory structure-function course for first-year students [5]. Frequent attendees earned higher scores on the comprehensive examination and higher final scores than those of their sporadic attendee counterparts [5]. The databank used by us did not allow for this analysis, but the frequency of attendance is certainly a point that needs to be evaluated for students with lower scores in several courses.

Predicting the academic performance of medical students is an object of intense interest. It has been shown that 23% of variance in medical school performance can be explained by previous academic performance and 6% of variance in postgraduate performance can be explained by previous academic performance [2]. Many educators consider the US Medical College Admissions Test (MCAT) to be the “gold standard” for predicting success in medical school [6], [7]. However, in a different study, MCAT scores did not demonstrate a significant correlation with the final score, pretest scores, or first-year through second-year score averages [5]. Furthermore, correlations of the admissions measures in MCAT with clinical performance during graduation were quite weak and couldn't be a reliable predictor of clinical skills [8]. The Graduate Australian Medical School Admissions Test (GAMSAT) has also been considered a poor predictor of academic performance [9]. As mentioned above, performance on admission tests does not seem to be a good predictor of medical students' performance during their studies. In this context, there is also evidence that the language and the pre medical school educational system can influence the student's performance in both admission tests and during the medical school [10]. The present report does not address the issue of selecting students for medical schools. We also did not address the issue of predicting professional capacity based on academic performance, which is also a relevant problem [11], [12]. Our approach was focused on possible advantages of an early identification of individuals who may benefit from special educational measures. Gauging the performance of medical students at the beginning of their medical education can provide better parameters to predict their yield during the remainder of their studies.

Teaching methods and pedagogical arrangement might not be uniformly efficient and may unequally benefit some types of students, preventing them from reaching their maximal potential. Low student performance could be correlated with poor study skills. Learning style covers both motivations for learning and the processes by which the student approaches the task of learning [2]. An individualized approach can help otherwise capable students to overcome their learning difficulties.

Another point to consider is the reported significant positive association between the use of strategic learning and final scores [13], [14], [15], as well the association of a consistent learning with performance in examinations [15], [16]. Additionally, students who emphasize the deductive method tend to perform better than those with any other style [13], [17]. It may therefore be useful for medical educational programs to inform students how to successfully use their study skills [18], [19]. Students in both groups, high and low performance, should be evaluated to verify if there are differences in their methods of studying and learning. Stimulating and encouraging students in the low-performance group to seek a more efficient method of studying can be of great help, as seen with peer-assisted learning, which motivates the learner to spend more time preparing, possibly resulting in deeper learning [20].

As demonstrated here, the overall performance of medical students in the first semester can predict their performances in subsequent semesters with a considerable relative risk (3.907 for the second and 2.873 for the eighth semester). To validate this observation, ROC curves were built using scores from the first semester and the performance outcomes in the following semesters. The analysis using ROC curves led us to identify a high cut-off score (7.188) in the first semester that was able to differentiate students with high performance from the ones with low performance during further semesters of medical school. This fact demonstrates that the medical students included in our sample had an overall good performance. One of the goals of medical school is to encourage students to develop a maximum theoretical learning ability and practical performance. A cut-off value can help the departments of the medical schools to identify those students who need special pedagogical intervention. Nevertheless, a cut-off value does not necessarily establish the capability or quality of their professional future.

Another aspect to highlight is the possibility of monitoring course scores, as mean and deviation, over a period of time. If the students' assessment in a course is monitored over time, the teachers and coordinators can discriminate between desirable and unwanted changes. We have shown that some courses have a constant increasing mean score over time, suggesting either a gradual softening in assessment, or a benefit of a maintained increase in the quality of teaching. In other courses, the students' average is always in the narrow range (±1SD of global media). This trend may be seen as a constant and equitable pattern of assessment, but it may also reflect the lack of a policy for improvement from the teachers. A marked oscillation of data around a global average suggests that there are periods with milder and others with more rigorous evaluations, as well as the absence of a steady teaching pattern. In any case, it is clear that courses must follow a stringent standard, maintain a good quality of teaching and assessment of the students, and observe time trends as a means to help implement adequate changes.

This study presents some limitations. The course content and assessment difficulty may have changed over the 10-year period. It limits the grouping of scores from students of different semesters. Performance of the students in course examinations often does not reflect their ability and professional skill. Thus, performance in the first semester is just a predictor of student performance in the rest of the theoretical part of the course and does not reflect necessarily their future medical performance.

Our study has at least two implications. Firstly, students' low performance in courses offered in the first semester of course is an acceptable parameter to predict their performance in subsequent courses. We propose that students with low performance at the beginning of their course merit special attention to minimize the risks of a continuous limitation. Secondly, an evaluation of the time trend of scores conferred by courses may help departments to monitor changes in personnel and methodology that may affect a student's performance.
